# The Eukaryotic-Type Serine/Threonine Protein Kinase Stk Is Required for Biofilm Formation and Virulence in *Staphylococcus epidermidis*

**DOI:** 10.1371/journal.pone.0025380

**Published:** 2011-09-23

**Authors:** Qian Liu, Jiajia Fan, Chen Niu, Decheng Wang, Jianping Wang, Xing Wang, Amer E. Villaruz, Min Li, Michael Otto, Qian Gao

**Affiliations:** 1 Key Laboratory of Medical Molecular Virology, Institutes of Biomedical Sciences, and Institute of Medical Microbiology, Fudan University, Shanghai, People's Republic of China; 2 National Institute of Allergy and Infectious Diseases, The National Institutes of Health, Bethesda, Maryland, United States of America; 3 Department of Laboratory Medicine, Huashan Hospital, Shanghai Medical College, Fudan University, Shanghai, People's Republic of China; University of Edinburgh, United Kingdom

## Abstract

**Background:**

Serine/threonine kinases are involved in gene regulation and signal transduction in prokaryotes and eukaryotes. Here, we investigated the role of the serine/threonine kinase Stk in the opportunistic pathogen *Staphylococcus epidermidis*.

**Methodology/Principal Findings:**

We constructed an isogenic *stk* mutant of a biofilm-forming clinical *S. epidermidis* isolate. Presence of *stk* was important for biofilm formation *in vitro* and virulence in a murine subcutaneous foreign body infection model. Furthermore, the *stk* mutant exhibited phenotypes indicating an impact of *stk* on metabolic pathways. Using different constructs for the genetic complementation of the *stk* mutant strain with full-length Stk or specific Stk domains, we determined that the Stk intracellular kinase domain is important for biofilm formation and regulation of purine metabolism. Site-specific inactivation of the Stk kinase domain led to defective biofilm formation, in further support of the notion that the kinase activity of Stk regulates biofilm formation of *S. epidermidis*. According to immunological detection of the biofilm exopolysaccharide PIA and real-time PCR of the PIA biosynthesis genes, the impact of *stk* on biofilm formation is mediated, at least in part, by a strong influence on PIA expression.

**Conclusions:**

Our study identifies Stk as an important regulator of biofilm formation and virulence of *S. epidermidis*, with additional involvement in purine metabolism and the bacterial stress response.

## Introduction

Reversible protein phosphorylation plays a fundamental role in signal transduction pathways in pro- and eukaryotes [1]. Various protein kinases mediate protein phosphorylation, which is generally coupled to dephosphorylation reactions catalyzed by protein phosphatases and enables translation of extracellular signals into cellular responses [2]. Since the first characterization of a eukaryote-like serine/threonine kinase (ESTK) in *Myxococcus xanthus*, similar ESTKs have been identified in numerous bacteria [3], [4], [5]. In particular, the Stk serine/threonine kinase has emerged as a critical signaling molecule in prokaryotes. In several bacterial species, Stk and similar proteins have been implicated in various phenotypes, including biofilm formation, cell wall biosynthesis, stress responses, metabolic pathways, autolysis and virulence [6], [7]. For example, a PknB-like sensor kinase with similarity to Stk proteins was found essential for growth in *Mycobacterium tuberculosis* [8] and further Stk-like proteins were reported to be involved in biofilm formation in *Streptococcus mutans*, *Bacillus subtilis* and *Mycobacterium smegmatis* [9], [10], [11]. Furthermore, Stk was shown to play a role in virulence in streptococci, *M. tuberculosis*, *Yersinia pseudotuberculosis* and *Staphylococcus aureu*s [12], [13], [14], [15]. Hence, Stk homologues are widely distributed in bacteria and involved in diverse steps of bacterial pathogenesis.

The opportunistic pathogen *Staphylococcus epidermidis*, a member of the coagulase-negative staphylococci group, colonizes the skin and mucous membranes of the human body [16]. Unlike *S. aureus*, which produces a series of aggressive virulence determinants and frequently causes severe acute infections, *S. epidermidis* mainly causes persistent infections associated with biofilm formation on indwelling medical devices [17]. With the increasingly frequent application of such devices in recent years, *S. epidermidis* has drawn substantial interest as one of the most common causes of nosocomial infections [18].

Biofilms are multilayered, surface attached agglomerations of microorganisms which have intrinsic resistance to host immune defenses and antibiotic treatment. *S. epidermidis* biofilm formation occurs in two stages, including initial attachment to a surface and subsequent accumulation of cells, leading to multicellular structures [19]. Several factors have been identified to play a significant role in biofilm formation of *S. epidermidis*. Determinants that affect primary attachment to abiotic surfaces include the bifunctional adhesin and autolysin AtlE, the biofilm associated protein Bap, the fibrinogen-binding protein Fbe/SdrG and the fibronectin-binding protein Embp [20], [21], [22], [23]. Polysaccharide intercellular adhesin (PIA) is crucial for biofilm formation *in vitro* and biofilm-associated infections [24]. In particular in PIA-negative *S. epidermidis*, the accumulation-associated protein (Aap) serves as an intercellular adhesin [25], [26]. Other factors, including the ClpP protease, the DNA-binding protein SarZ, the substrate of ClpP, Spx, and extracellular DNA have also been reported to be directly or indirectly impact *S. epidermidis* biofilm formation [27], [28], [29], [30].

Biofilm formation in *S. epidermidis* is under the influence of a diverse range of regulatory mechanisms [17]. For example, the quorum-sensing system *agr* (accessory gene regulator) regulates adhesin factors during the early stage of biofilm formation [31]. The *icaADBC* locus, encoding the proteins responsible for the synthesis and deacetylation of PIA, is up-regulated by the global regulator sigma factor SigB [32]. The product of the *icaR* gene, located adjacent to the *ica* operon, is a negative regulator of the *icaADBC* operon [33]. However, the signaling networks that control *S. epidermidis* biofilm formation remain incompletely understood.

In the present study, we identified a putative ESTK protein-encoding gene (designated *stk*) and a co-transcribed eukaryotic-like serine/threonine phosphatase gene (designated *stp*) in *S. epidermidis*. To explore the function of Stk in *S. epidermidis*, we constructed and characterized an *stk* mutant strain, which revealed important roles of Stk in biofilm formation, virulence, and metabolism of *S. epidermidis*.

## Results

### Genome context of *stk* and *stp* in *S. epidermidis*

**Figure 1:**
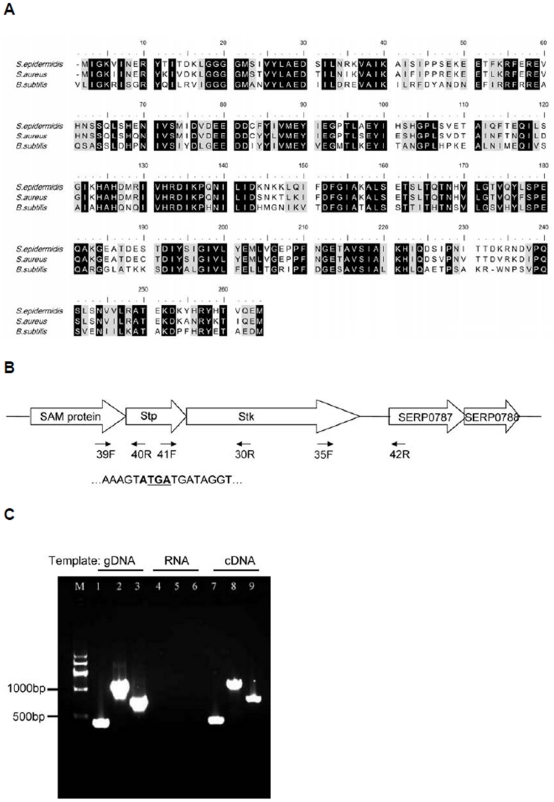
The genomic context of *stk* and *stp* in *S. epidermidis*. **A.** Alignment of *stk* sequences of *S. epidermidis* 1457 with those of *S. aureus* and *B. subtilis*, showing conserved amino acid positions at the bottom. **B.** Schematic of the *stk/stp* genetic locus showing primer annealing sites. The four nucleotides by which the two genes overlap are in bold. The stop codon for *stp* is underlined. **C.** Co-transcription analysis of the four genes SERP0784 to SERP0787 using reverse transcription (RT-) PCR analysis with genomic DNA (gDNA), RNA or cDNA as templates. Lanes 1, 4, and 7 represent the amplification using primer set 39F and 40R, lanes 2, 5, and 8 represent the amplification using primer set 41F and 30R, lanes 3, 6, and 9 represent the amplification using primer set 35F and 42R.

**Figure 2:**
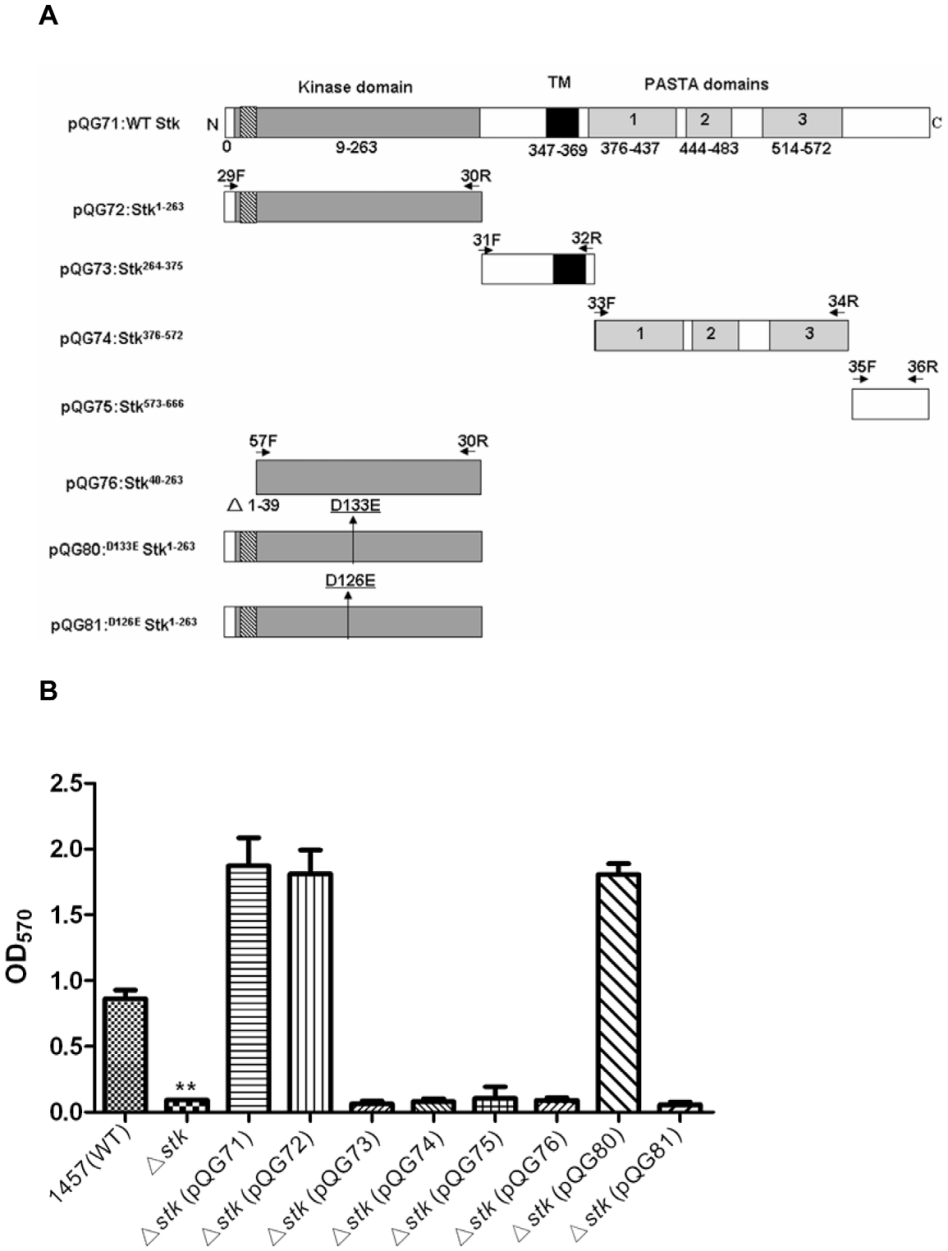
Stk influences biofilm formation *in vitro*. **A.** Architecture of *stk* complementation constructs. Slashed lines denote the ATP-binding site of Stk. The primers used for the amplification of specific domains are indicated by arrows. TM, transmembrane. **B.** Influence of Stk and Stk domains on *S. epidermidis* biofilm formation. The *stk* mutant strain was complemented with plasmids expressing different domains of Stk: pQG71, full length; pQG72, kinase domain; pQG73, transmembrane domain; pQG74, PASTA repeated domain; pQG75, C-terminal domain; pQG76, kinase domain without ATP-binding site; pQG80, kinase domain with D133E mutation; pQG81, kinase domain with D126E mutation, and biofilm formation in comparison to the *stk* mutant and WT strains was determined *in vitro* by semiquantitative biofilm assays. Data were derived from three independent experiments, with 4 replicates in each experiment. **, P<0.01 (versus WT).

ESTKs have been identified in a wide range of prokaryotes. We performed a genome search (using BlastP, http://blast.ncbi.nlm.nih.gov/Blast.cgi) of *S. epidermidis* strain RP62A, which revealed the presence of a gene, SERP0786, whose protein product shows pronounced homology to Stk proteins in other species (36% identity with PrkC of *B. subtilis* and 67% identity with Stk of *S. aureus*) (Fig. 1A). We therefore designated the gene *stk*. The 3′ end of *stk* overlaps with the adjacent gene, which encodes a protein phosphatase (SERP0785, designated *stp*) (Fig. 1B). Thus, *stk* and *stp* are likely co-transcribed [34], which we confirmed by reverse transcription (RT-) PCR analysis using primers 41F and 30R, one of which hybridizes with a sequence within the *stk* and the other with one within the *stp* gene. RT-PCR analysis also demonstrated co-transcription of *stp* and *stk* with the adjacent up- and downstream genes, encoding a SAM protein and a hypothetical protein, respectively (Fig. 1C). Of note, the 666-amino acid (aa) Stk of *S. epidermidis* displays conserved motifs that fit the description of a Hank's-type ESTK [35]: it includes an intracellular ATP-binding kinase domain (9–263 aa), a transmembrane spanning domain (347–369 aa) and three extracellular PASTA (penicillin and Ser/Thr kinase associated) repeated domains (376–437 aa, 444–483 aa, 514–572 aa) [36]. Within the N-terminal catalytic kinase domain, the protein harbors an important ATP-binding site (16–39 aa) that is presumably essential for kinase activity (Fig. 2A).

### Inactivation of *stk* in *S. epidermidis* results in reduced biofilm formation *in vitro*

To investigate the role of Stk in *S. epidermidis* physiology and pathogenesis, we constructed an isogenic deletion mutant of the *stk* gene in the biofilm-forming clinical isolate *S. epidermidis* 1457. Then, to determine whether *stk* influences biofilm formation as a main virulence phenotype in *S. epidermidis*, we analyzed biofilm formation by a semi-quantitative colorimetric assay. We found that in-vitro biofilm formation of the *stk* mutant strain was significantly impaired compared to the WT strain (Fig. 2B). The impact of *stk* on biofilm formation was confirmed by genetic complementation with the entire copy of *stk* expressed from a plasmid (Δ*stk* (pQG71)) (Fig. 2B). The complemented strains showed much higher biofilm-forming capacity compared to the WT, which is likely mainly due to a plasmid copy effect and the use of a strong promoter (*ica*) to express the *stk* gene.

**Figure 3:**
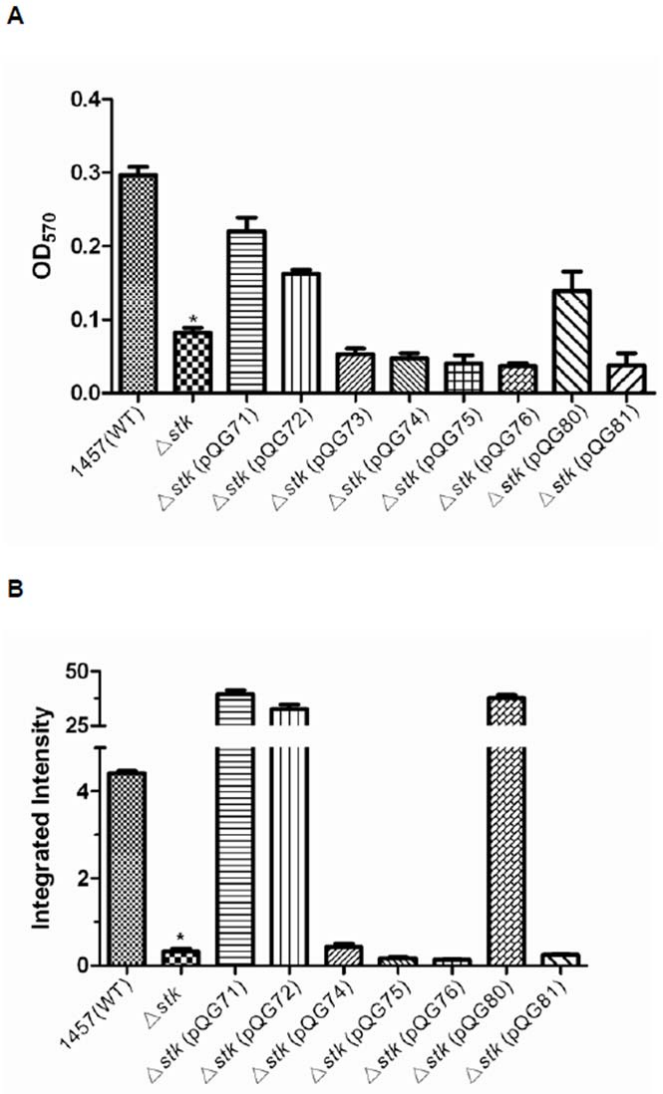
Stk influences primary attachment and PIA production *in vitro*. **A.** Influence of Stk and Stk domains on primary attachment. The *stk* mutant strain was complemented with plasmids expressing different domains of Stk: pQG71, full length; pQG72, kinase domain; pQG73, transmembrane domain; pQG74, PASTA repeated domain; pQG75, C-terminal domain; pQG76, kinase domain without ATP-binding site; pQG80, kinase domain with D133E mutation; pQG81, kinase domain with D126E mutation, and primary attachment was determined. Data were derived from three independent experiments, with 4 replicates in each experiment. *, P<0.05 (versus WT). **B.** Influence of Stk and Stk domains on polysaccharide intercellular adhesin (PIA) production. PIA was determined in the same strains as in (A) using immunodot blots with anti-PIA serum and photodigital evaluation. Values represent the means ± standard errors of the means from three independent experiments, *, P<0.05 (versus WT).

To study which stage of biofilm formation is influenced by *stk*, we performed a primary attachment assay and found that the *stk* mutant strain displayed decreased attachment ability to polystyrene compared with the WT strain, while this ability was restored in the complemented mutant strain (Fig. 3A). Furthermore, immunoblot analysis of polysaccharide intercellular adhesin (PIA), a key factor contributing to biofilm accumulation of *S. epidermidis* [37] demonstrated that *stk* positively affects PIA production and thus the second stage of biofilm formation (Fig. 3B). These observations indicate that *stk* is an important regulator of *S. epidermidis* biofilm formation in both the initial and accumulative stages.

### The kinase activity of Stk is crucial for biofilm formation in *S. epidermidis*

To determine which domains of the Stk protein are important for its influence on biofilm formation, we genetically complemented the *stk* mutant strain with constructs expressing different Stk domains (Fig. 2A). Only the strain expressing the kinase domain (Δ*stk* (pQG72)) restored biofilm formation to a level comparable to that of the WT, whereas the strains expressing only the transmembrane segment (Δ*stk* (pQG73)), the PASTA repeated domain (Δ*stk* (pQG74)) or the C-terminal segment (Δ*stk* (pQG75)) did not form a biofilm. Similarly, a strain expressing a truncated kinase domain lacking the conserved ATP-binding site (Δ*stk* (pQG76)) also did not form biofilm (Fig. 2B).

As it has been reported that the conserved aspartic acid residue D_126_ is crucial for the catalytic activity of the enzyme [13], we confirmed the role of the kinase activity in biofilm formation in *S. epidermidis* using a D126E mutant (Δ*stk* (pQG81)). In addition, we mutated the D_133_ residue that is in close proximity to D_126_ to investigate the specificity of the effect exerted by the D126E exchange. The D126E mutant showed significantly decreased biofilm formation compared to the clone expressing the unaltered kinase domain, while biofilm formation of the D133E mutant (Δ*stk* (pQG80)) was not changed (Fig. 2B). Corresponding results were achieved in the primary attachment and PIA production assays (Fig. 3). These findings indicated that the kinase activity of Stk is crucial for its impact on primary attachment, PIA production, and biofilm formation.

### The predicted kinase domain of *S. epidermidis* Stk has kinase activity

**Figure 4:**
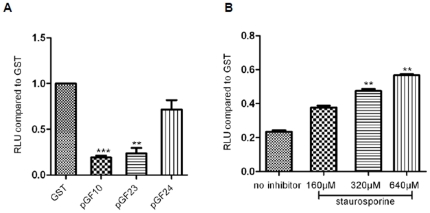
The kinase activities of GST fusion proteins were measured *in vitro*. **A.** Phosphorylation activities of GST fusion proteins containing full-length Stk (using plasmid pQG77), Stk^1–263^ (pQG78) or Stk^40–263^(pQG79) were measured with a Kinase-Glo™ luminescent kinase assay *in vitro* at 32°C for 4 h. **B.** Phosphorylation activities of GST fusion proteins containing full-length Stk (using plasmid pQG77) were measured with a Kinase-Glo™ luminescent kinase assay with or without staurosporine *in vitro* at 32°C for 4 h. GST was used as a negative control. RLU, relative luminescent unit. Data are derived from three independent experiments, **, P<0.01; ***, P<0.001 (versus GST).

To further confirm the involvement of the kinase domain in kinase activity, we produced GST fusion constructs with the entire Stk protein, only the kinase domain, or an inactivated kinase domain, in which the ATP-binding cassette was deleted. In kinase assays, only the GST fusion construct with the kinase domain lacking the ATP binding cassette did not exhibit activity, showing that the cassette is crucial for kinase activity (Fig. 4A). Staurosporine, a potent protein kinase inhibitor, inhibited the WT Stk activity in a dose-dependent manner, showing significant inhibition at a concentration of 320 µM and above (Fig. 4B).

### Effect of the *stk* mutation on the transcription of biofilm-related genes

**Figure 5:**
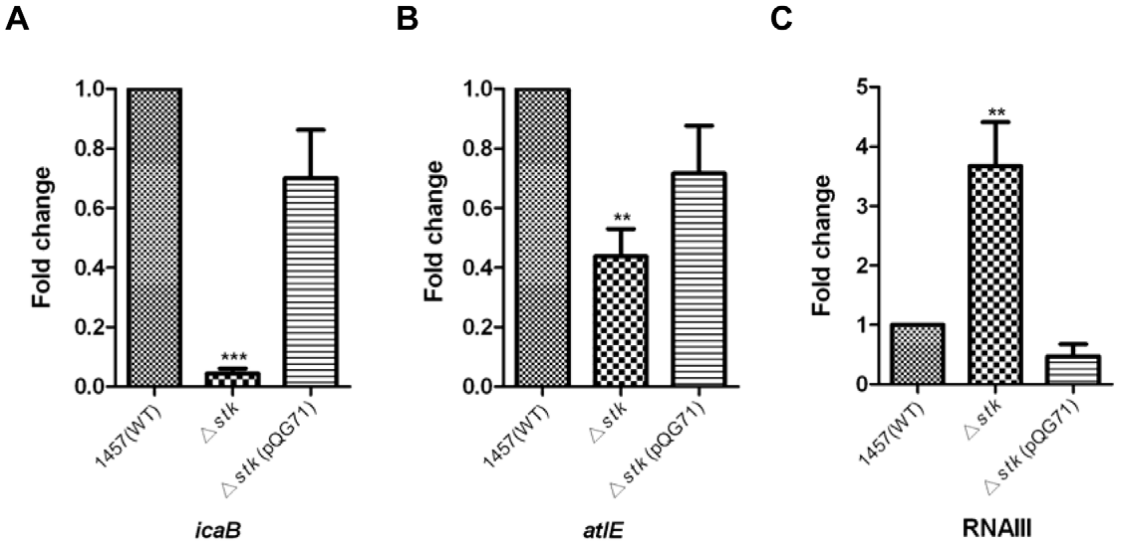
Influence of Stk on the expression of the biofilm-associated genes. The *stk* mutant strain was complemented with plasmids expressing Stk full length (pQG71) and expression of the *icaB* (to monitor expression of the *icaADBC* operon), RNAIII and *atlE* genes was determined in samples prepared from exponential-phase (OD_600_ = 0.5) cells grown in BM, using quantitative RT-PCR. Data were derived from three biological repeats. **, P<0.01; ***, P<0.001(versus WT).

ESTKs commonly serve as global regulators of gene expression [38]. To analyze whether the impact of *S. epidermidis* Stk on biofilm formation is mediated by altering expression of biofilm-related regulatory or structural genes, we tested the transcriptional levels of several such genes in the *stk* mutant and wild-type strains by quantitative RT-PCR. We detected a significant positive impact of *stk* on the expression of the *icaB* and *atlE* genes, as expected from our results showing increased PIA production and primary attachment, of which AtlE is a key determinant [21] (Fig. 5A&B). To examine whether *stk* has an impact on the expression of global regulatory system, we examined transcript levels of *sarA* and *agr*, the latter by measuring RNAIII. The transcript levels of *agr* were up-regulated significantly in the mutant strain compared with WT strain (Fig. 5C). In contrast, *stk* did not impact expression of *sarA* (data not shown). Interestingly, we also did not detect an impact on the expression of the *icaR* regulatory gene (data not shown), indicating that the *icaADBC* but not the *icaR* promoter is under the influence of *stk*-dependent regulation.

### The role of *stk* in the pathogenesis of *S. epidermidis* biofilm-associated infection

**Figure 6:**
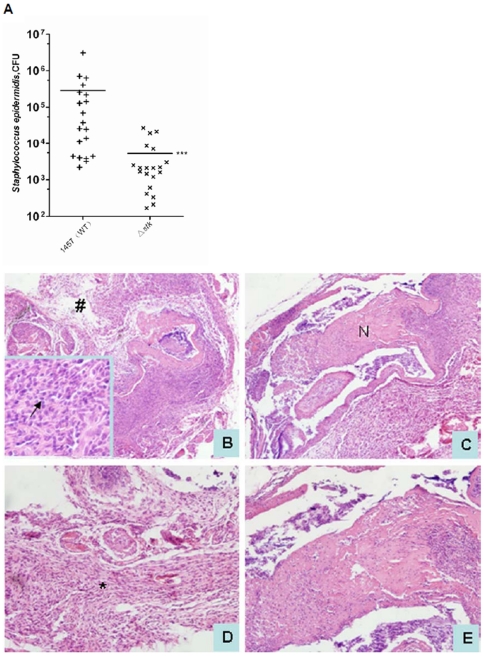
Murine model of subcutaneous catheter infection. **A.** Catheters were inserted into the subcutaneous tissue of mice, followed by the injection of 1×10^7^ CFU of the *S. epidermidis* 1457 WT or *stk* mutant strain. After 10 days, catheters were removed from all mice and CFUs of recovered bacteria on the catheters were counted. Data were analyzed using a Mann-Whitney test. The horizontal line represents the median. ***, P<0.001. **B–E.** Histopathological analysis of skin tissues around catheters infected with either *S. epidermidis* 1457 wild-type (**B, D**) or its isogenic *stk* mutant (**C, E**). **B, C**: Low magnification (100×); **D, E**: High magnification (200×). * denotes disseminated inflammatory cells, **#** denotes disrupted structure, **N** denotes normal structure. The black arrow in the panel B insert (400× magnification) indicates infiltrating neutrophils.

To investigate the impact of *stk* on biofilm-associated infection *in vivo*, we performed a murine subcutaneous foreign body infection model. Progression of disease was measured by determining bacterial loads on the implanted catheters after sacrifice of the test animals. We detected a significantly lower bacterial load in animals infected with the *stk* mutant compared to those infected with the WT strain (Fig. 6A), indicating that *stk* contributes to pathogen survival during *S. epidermidis* infection. Furthermore, histopathological analysis demonstrated that inflammatory lesions were stronger in the epithelial tissue around the implanted device of the mice infected with the WT strain compared to those infected with the mutant strain (Fig. 6B–E). Fibrous structures of epithelial tissues were severely damaged and numerous inflammatory cells were found that had infiltrated into the infected tissues in the WT group. Among those cells, neutrophils were the predominant type by microscopic analysis. In contrast, in the epithelial tissues of mutant-infected mice only mild inflammatory responses (few infiltrated inflammatory cells and slight tissue lesions) were observed. These results indicated that *stk* plays a significant role in biofilm-associated infection in *S. epidermidis*.

### Phenotypic analysis of an isogenic *S. epidermidis stk* deletion mutant

**Figure 7:**
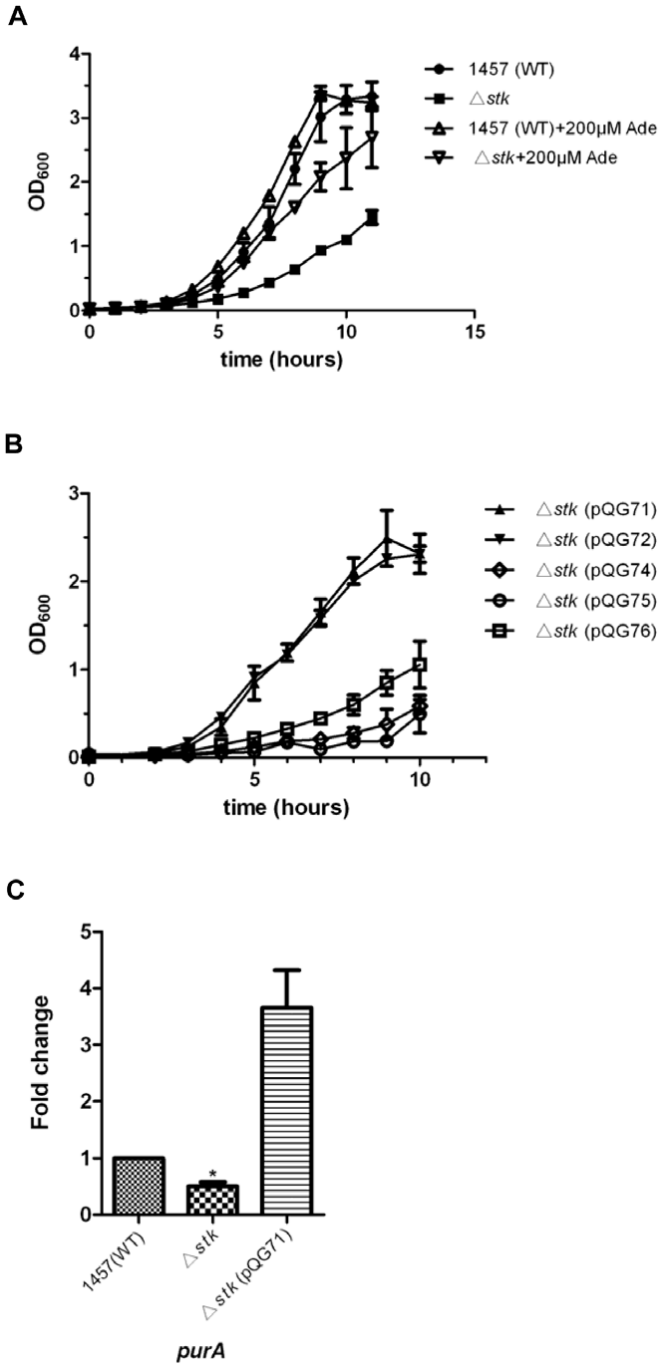
Stk is involved in purine biosynthesis in *S. epidermidis*. **A.** Growth curve of *S. epidermidis* 1457 WT and the isogenic *stk* mutant. Growth was determined by measuring OD_600_ of cell cultures grown in RPMI 1640 synthetic medium at 37°C with or without addition of 200 µM adenine (Ade). **B.** Growth curves of *stk* mutant strains with different complementation plasmids: pQG71 (*stk* full length), pQG72 (*stk* kinase domain), pQG74 (*stk* PASTA repeated domain), pQG75 (*stk* C-terminal domain), pQG76 (*stk* kinase domain without ATP-binding site). Cell growth was monitored at OD_600_ in RPMI 1640 synthetic medium at 37°C. **C.** Expression levels of *purA* was determined in samples prepared from exponential-phase (OD_600_ = 0.5) cells grown in BM, using quantitative RT-PCR. Data were derived from three biological repeats. *, P<0.05 (versus WT).

We then analyzed whether Stk has an influence on metabolic pathways, as these phenotypes are reportedly under Stk control in other bacteria [7], [39], [40]. Growth of the isogenic *stk* mutant in TSB was similar to that of the WT strain (data not shown). However, in defined RPMI 1640 medium growth of the *stk* mutant strain was slower compared to the WT strain, while supplementing the medium with 200 µM adenine partially reversed the growth defect of the *stk* mutant strain (Fig. 7A). Interestingly, in addition to full-length Stk, the Stk kinase domain alone was able to complement the growth defect of the *stk* mutant, in contrast to the PASTA domains or an inactivated kinase domain (Fig. 7B). To analyze whether the impact of *S. epidermidis* Stk on purine biosynthesis is mediated by altering expression of purine-related regulatory genes, we tested the transcriptional levels of several such genes in the *stk* mutant and WT strains by quantitative RT-PCR. We detected a significant positive impact of *stk* on the expression of the *purA* gene (Fig. 7C). In contrast, *stk* did not impact expression of *purR* (data not shown). These results indicated that Stk in *S. epidermidis* is involved in purine biosynthesis through the kinase activity, maybe by regulating the expression of *purA* gene.

**Figure 8:**
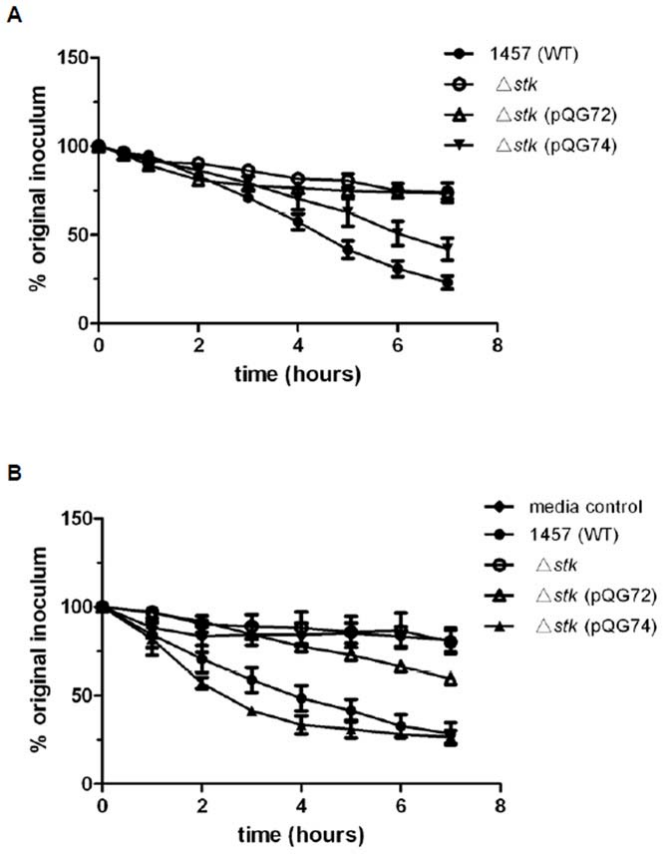
Impact of Stk on cell wall-related phenotypes. **A.** Triton X-100-induced autolysis of *S. epidermidis* strains. Cells of the WT, the *stk* mutant, and the mutant complemented with the kinase domain (Δ*stk* (pQG72)), or PASTA repeated domain (Δ*stk* (pQG74)), were treated with 0.1% Triton X-100. **B.** Autolysin activity. Bacteriolytic activities of secreted autolysin in the supernatants of stationary phase cultures of the WT, the *stk* mutant and the complemented strains (Δ*stk* (pQG72) and Δ*stk* (pQG74)), were determined using heat-killed *S. epidermidis* 1457 WT cells as the substrate. TSB was used as media control.

Furthermore, the *stk* mutant was more resistant to Triton X-100 induced lysis than the WT strain, which could be complemented with the PASTA, but not the kinase domain (Fig. 8A). Moreover, culture filtrate of the *stk* mutant strain exhibited diminished autolytic activity, which also could be complemented with the PASTA but not the kinase domain (Fig. 8B). These results indicated that Stk in *S. epidermidis* is involved in the bacterial stress response.

## Discussion

In this study, we characterized the genetic locus encoding the ESTK-like Stk protein of *S. epidermidis* and report on the roles of this gene in physiology and virulence. Similar to *S. aureus* and *Streptococcus pyogenes* [34], [39], the *stk* gene is adjacent to, and as we show here co-transcribed with, the *stp* gene encoding the cognate phosphatase. Deletion of *stk* did not have an effect on the transcription of *stp*, as shown by quantitative RT-PCR (data not shown). Since biofilm formation is a crucial mechanism by which *S. epidermidis* escapes human host defenses, we determined whether *stk* contributes to biofilm formation in *S. epidermidis*. Our data show that *stk* is involved in both the initial attachment and subsequent accumulation stages of biofilm formation and this impact is mediated at least in part by altering expression of the global regulator *agr*, *atlE* genes and the *ica* operon, the latter resulting in increased production of the biofilm exopolysaccharide PIA. Likely as a consequence of the impact of *stk* on these biofilm- and immune evasion-related genes, we also detected a significantly decreased virulence potential of the *stk* mutant strain in a murine subcutaneous foreign body infection model. This is consistent with reports on the in-vivo role of Stk in other prokaryotes such as *S. aureus*, *S. pneumoniae* and *M. tuberculosis* [12], [41], [42]. Of note, the effect of *stk* on *S. epidermidis* pathogenesis may be largely dependent on the strong regulation of PIA synthesis, since PIA acts both as a matrix and contributes to immune evasion [43], [44], [45].

Similar to other serine/threonine kinases in Gram-positive bacteria, the Stk protein of *S. epidermidis* has two domains (four segments), including an amino-terminal kinase domain, a transmembrane segment, an extracellular PASTA domain (three PASTA repeats) and a carboxyl-terminal part [46]. We showed here that the kinase domain, whose kinase activity we confirmed, is crucial for the impact of Stk on the detected in-vitro biofilm phenotype.

According to our results, *S. epidermidis* Stk is also involved in the regulation of metabolism. In particular, delayed growth in RPMI medium, which could be overcome by adding adenine and genetic complementation with the kinase domain, indicated an impact of Stk on purine metabolism that is dependent on the Stk kinase domain. Our results are consistent with previous reports showing that a mutant of the purine biosynthesis gene *purR* of *S. epidermidis* displayed lower PIA production and was biofilm-negative [47], [48] and a study that showed regulation of purine biosynthesis by the ESTK protein PknB of *S. aureus*, which regulates the adenylosuccinate synthase PurA via reversible phosphorylation [38]. It has been reported that kinase-dependent phosphorylation decreases PurA activity and is responsible for regulating purine biosynthesis in *Streptococcus agalactiae* [49], which seems to contrast our results. However, it is conceivable that PurA activity is regulated at the transcriptional and/or translational levels in different organisms. How exactly PurA function is regulated in *S. epidermidis* remains to be investigated.

Furthermore, we showed that Stk had a significant influence on the bacterial stress response. The influence was mainly dependent on the extracellular PASTA domains, which are highly conserved and comprise three beta sheets and an alpha helix that binds the beta-lactam stem [50]. Stk proteins containing PASTA domains have been shown to play a key role in cell wall biosynthesis and suggested to sense dissociated peptidoglycan subunits [13], [36], [51]. It is likely that the PASTA repeats of the *S. epidermidis* Stk protein also recognize unlinked peptidoglycan and binding of those structures activates the Stk cytoplasmic N-terminal kinase domain.

In conclusion, to our knowledge this is the first study to show that Stk influences biofilm formation through its kinase activity and has an impact on virulence in *S. epidermidis*. Additionally, we showed that *stk* is involved in purine biosynthesis and stress response in *S. epidermidis*. While it is likely that Stk exerts its regulatory function directly through phosphorylation of proteins involved in biofilm formation or metabolism, with several such Stk substrates having been identified in other organisms to date [34], [52], [53], the specific substrates of Stk in *S. epidermidis* remain to be determined.

## Materials and Methods

### Ethics Statement

Forty BALB/c mice were bought from Laboratory Animal Center of Fudan University. All animal work was approved by the Institutional Animal Care and Use Committee (IACUC), Fudan University Shanghai Medical College. IACUC Animal Project Number (APN): 20061203-gao.

### Bacterial strains and growth conditions

The bacteria and plasmids used are listed in Table 1. *Escherichia coli* DH5α and BL21 DE3 pLysS were grown in Luria-Bertani (LB) medium. Plasmid-containing *E. coli* strains were grown in the same medium but with ampicillin (100 µg/ml) or kanamycin (20 µg/ml) included, as appropriate. *S. aureus* RN4220 was used as a gateway strain for plasmid transformation into target *S. epidermidis* strains. *S. epidermidis* and its derivative strains were cultured in tryptic soy broth (TSB, Oxoid) or basic medium (BM: 1% (wt/vol) trypton, 0.5% (wt/vol) yeast extract, 0.1% (wt/vol) glucose, 85 mM NaCl, 4 mM K_2_PO_4_), and when necessary, erythromycin (10 µg/ml), chloramphenicol (10 µg/ml), and anhydrous tetracycline (1 µg/ml) were supplemented. Media were solidified with 1.5% (wt/vol) agar as needed.

**Table 1:** Bacterial strains and plasmids used in this study.

Strain or plasmid	Description^a^	Source or reference
Strains		
*S. epidermidis* 1457	Clinical isolate, biofilm positive	[62]
*S. aureus* RN4220	Clining host, restriction negative, modification positive	[63]
*E. coli* DH5α	*supE44 ΔlacU169* (Φ80d*lacZ*ΔM15) *hsdR17 recA1 endA1 gyrA96 thi-1 relA1*	Invitrogen
*E. coli* BL21 DE3 pLysS	*E. coli B F- omp*T *hsd*SB(rB-, mB-) *gal dcm* (DE3) pLysS CamR	Invitrogen
Plasmids		
pKOR1	Shuttle vector, temperature sensitive, vector for allelic replacement via lambda recombination and *ccdB* selection Cm^R^ Amp^R^	[56]
pKOR1*stk*	pKOR1 harboring the *stk* gene, Cm^R^ Amp^R^	This study
pYJ90	Shuttle vector, Amp^R^ Erm^R^	[64]
pQG70	pYJ90 harboring the promoter sequence of *icaADBC*, Amp^R^ Erm^R^	This study
pQG71	pYJ70 harboring the full-length *stk* gene, Amp^R^ Erm^R^	This study
pQG72	pYJ70 harboring the *stk* gene kinase domain, Amp^R^ Erm^R^	This study
pQG73	pYJ70 harboring the *stk* gene transmembrane domain, Amp^R^ Erm^R^	This study
pQG74	pYJ70 harboring the *stk* gene three repeated PSATA domains, Amp^R^ Erm^R^	This study
pQG75	pYJ70 harboring the *stk* gene C-terminal domain, Amp^R^ Erm^R^	This study
pQG76	pYJ70 harboring the *stk* gene kinase domain without ATP-binding site, Amp^R^ Erm^R^	This study
pQG80	pYJ70 harboring the *stk* gene kinase domain with an Asp to Glu exchange at position 133, Amp^R^ Erm^R^	This study
pQG81	pYJ70 harboring the *stk* gene kinase domain with an Asp to Glu exchange at position 126, Amp^R^ Erm^R^	This study
pET28a	*E.coli* expression plasmid, Km^R^	Novagen
pGEX-KG	*E.coli* expression plasmid, Amp^R^	Amersham
pQG77	pGEX-KG harboring full-length Stk, Amp^R^	This study
pQG78	pGEX-KG harboring the Stk kinase domain (Stk^1–263^), Amp^R^	This study
pQG79	pGEX-KG harboring the Stk kinase domain without ATP binding site (Stk^40–263^), Amp^R^	This study

a Cm^R^, chloramphenicol resistance; Amp^R^, ampicillin resistance; Erm^R^, erythromycin resistance; Km^R^, kanamycin resistance.

### DNA manipulation

Genomic DNA of *S. epidermidis* 1457 was prepared by a standard protocol for gram-positive bacteria [54]. Plasmid DNA from *E. coli* was extracted using a plasmid purification kit (Axygen). Plasmid DNA from *S. aureus* and *S. epidermidis* was extracted using the same kit except that the cells were incubated for at least 30 min at 37°C in solution I (P1, suspension buffer) with lysostaphin (0.8 µg/µl; Sigma) before solution II (P2, dissociation buffer) was added. *PrimStar* polymerase and restriction enzymes were obtained from New England Biolabs (NEB). S. *epidermidis* was transformed by electroporation as described previously [55].

### Co-transcription assay

At an OD_600_ value of 0.5, cells in 1.5-ml TSB cultures were harvested and resuspended in 1.3 ml Trizol (Invitrogen). Cells were disrupted by shaking with a Mini-Beadbeater (Biospec Products) at maximum speed for 30 s. Tubes were then incubated on ice for 5 min. This shaking/cooling cycle was repeated 4 times. Then, the suspension was centrifuged. Total RNA isolation from the supernatant was performed in Trizol (Invitrogen) according to the manufacturer's instructions. After treatment using a TURBO DNA-free™ kit (Ambion), approximately 2 µg total RNA was used to create cDNA using a PrimeScript RT reagent kit (TaKaRa). An identical reaction was performed without reverse transcriptase as a negative control. cDNA with or without reverse transcriptase and genomic DNA (gDNA) were used as templates in PCRs using specific primer sets specific for overlapping (41F/30R), and outermost regions of *stp* and *stk* (39F/40R and 35F/42R), as shown in Fig. 1B.

### Construction of an isogenic *stk* deletion mutant and complemented strains

To delete the *stk* gene in *S. epidermidis* 1457, ∼1-kb DNA fragments, upstream and downstream of *stk*, were amplified by PCR from the chromosomal DNA of *S. epidermidis* 1457 using the primers listed in Table 2, introducing an overlapping sequence of ∼15 bp. The resulting fusion PCR fragment with *attB* sites at both ends was used for recombination with plasmid pKOR1, yielding plasmid pKOR1*stk*. It was transferred via electroporation first to *S. aureus* RN4220 and then to *S. epidermidis* 1457. Allelic replacement and selection of positive clones were performed as described [56]. The integration site was verified by analytical PCR and sequencing. As the sequence and location of the endogenous promoter that facilitates *stk* transcription in *S. epidermidis* are unknown, we used the promoter sequence of the *icaADBC* operon for the construction of a genetic complementation plasmid. This fragment was amplified from *S. epidermidis* 1457 genomic DNA by PCR using the primer set Pica1 and Pica2 and cloned into *Eco*RI and *Xma*I sites of *Eco*RI/*Xma*I-digested pYJ90, yielding pQG70. Plasmid pQG71 (*stk* full length complementing plasmid) was constructed by cloning the entire coding region of *stk* (1998 bp) into *Bam*HI/*Xma*I-digested pQG70, using the primer set *stk*-F and *stk*-R. Plasmids pQG72 (*stk* kinase domain complementing plasmid), pQG73 (*stk* transmembrane domain complementing plasmid), pQG74 (*stk* PASTA repeated domain complementing plasmid), pQG75 (*stk* C-terminal domain complementing plasmid) and pQG76 (*stk* kinase domain without ATP-binding site complementing plasmid) were constructed by cloning the respective domains into *Bam*HI/*Xma*I-digested pQG70, using the following primers: 29F and 30R for the kinase domain, 31F and 32R for the transmembrane domain, 33F and 34R for the PASTA domain, 35F and 36R for the C-terminal domain, and 57F and 30R for the kinase domain without ATP-binding segment. All these plasmids (Table 1) were used to transform the *stk* mutant strain.

**Table 2:** Primers used in this study.

Primer	Sequence (5′-3′)^a^
SEAtt1	**GGGGACCACTTTGTACAAGAAAGCTGGGT**TACCGTCCATCACATCAAAG
SERev1	ACTTTATCACCTTCTAACTTTAAAAGGATA
SERev2	CTTTTAAAGTTAGAAGGTGATAAAGTATGAGTAACAACTAATGGGAAGTAGAT
SEAtt2	**GGGGACAAGTTTGTACAAAAAAGCAGGCT**GTAAAATTGTTTCTACAGATGTTCCGGGA
Stk-F	TCCC*CCCGGG*AGGAGGTGATAAAGTATGATAGGTAAA
Stk-R	CGT*GGATCC*CCCATTAGTTGTTACTCATTTAGCC
29F	TCCC*CCCGGG*AGGAGGATGATAGGTAAAGTCATAAATGAAC
30R	CGC*GGATCC*GCGTTACATTTCTTGAACAGTATGATA
31F	TACC*CCCGGG*AGGAGGATGTGTGACGATTTAACAAGTGCG
32R	CGCCGC*GGATCC*GCGCTATTCTTCATATTTATTTCC
33F	TACC*CCCGGG*AGGAGGATGCCTGATCTTAAAGGGAAA
34R	CGCCGC*GGATCC*GCGCTAAGAAACTACTAATAGTATAGT
35F	TCCC*CCCGGG*AGGAGGATGAAAGGAGAAAAGTCTGATGAA
36R	CGC*GGATCC*TTAGCCATCATAGCTCACATC
57F	ATAT*CCCGGG*AGGAGGATGGCGATTTCGATTCCTCCAAGT
39F	TGCCACGTTAATTTAATACCA
40R	CACGATTCAGCACGATTTATT
41F	GGGGCAAATTACTAAAGATGAAGC
42R	CGCCCACCACAGGTGAAAACTTC
17F	CCG*GAATTC*ATAGGTAAAGTCATAAATGAAC
18R	AAGGAAAAAA*GCGGCCGC*TTAGCCATCATAGCTCACATC
58F	AT*GAATTC*TGATGGCGATTTCGATTCCTCCAAGT
59F	CG*GAATTC*GGATGATAGGTAAAGTCATAAAT
60R	GCC*AAGCTT*TTACATTTCTTGAACAGTATGAT
43F	GCGCATGACATGAGAATTGTTCATCG***C***GA***G***ATTAAACCACAG
44R	CTGTGGTTTAAT***C***TC***G***CGATGAACAATTCTCATGTCATGCGC
53F	GAGTGGAATCAAACATGCGCATGA***G***ATGAGAATTGTTCA
54R	TGAACAATTCTCAT***C***TCATGCGCATGTTTGATTCCACTC
P*ica*1	CGG*GAATTC*AGTGCTTCTGGAGCACTAAAC
P*ica*2	CG*GGATCC*ACCTACCTTTCGTTAGTTAGG
RT-*gyrB*1	TGACGAGGCATTAGCAGGTT
RT-*gyr*B2	GTGAAGACCGCCAGATACTTT
RT-*atlE*1	GATGGATTGCTGCTAAGGATTT
RT-*atlE*2	TATCGGTTTGCTTTTGTTGG
RT-*icaB*1	GAAACAGGCTTATGGGACTTTG
RT-*icaB*2	CAAGTGCGCGTTCATTTTT
RT-*icaR*1	CATTGACGGACTTTACCAGTTTT
RT-*icaR*2	ATCCAAAGCGATGTGCGTAG
RT-*purA*1	GCATACGTAGATAAAGCACAA
RT-*purA*2	TGGACAAGTTTCGTTAAACAT
RT-*purR*1	ACTTGATGCTGTTGTTACCAT
RT-*purR*2	CCTTCTGTAACTTTATTGTCCT

a Incorporated restriction sites are in italic and underlined, *attB* sites are boldfaced and underlined, while the mutation sites are boldfaced and in italic.

### Site-directed mutagenesis

Site-directed mutagenesis was performed as described by Ho et al [57]. Briefly, a DNA fragment with the *stk* gene carrying a distinct single aspartic acid residue codon mutated to glutamic acid at position 133, or 126 was cloned into pQG70, yielding pQG80 and pQG81 respectively. The oligonucleotide primers 43F and 44R were used for mutagenesis at D_133_, while 53F and 54R were used for mutagenesis at D_126_. The presence of the mutation was verified by DNA sequencing. The two plasmids (Table 1) were used to transform the *stk* mutant strain.

### Semi-quantitative biofilm assay

Semi-quantitative biofilm assays were performed as described in our previous work [58]. Briefly, overnight cultures of *S. epidermidis* strains were diluted 1∶100 into fresh TSB. The diluted cultures were pipetted into sterile 96-well flat-bottom tissue culture plates and incubated at 37°C for 24 h. Culture supernatants were gently removed, and wells were washed with phosphate-buffered saline (PBS). The adherent organisms at the bottom of the wells were fixed by Bouin fixative over 1 h. Then the fixative was gently removed, wells were washed with PBS and stained with 0.4% (wt/vol) crystal violet. Biofilm formation was measured with a MicroELISA autoreader.

### Primary attachment assay

The assay was performed as described in our previous work [58]. Briefly, overnight cultures of *S. epidermidis* strains were diluted in fresh BM to an OD_600_ value of 0.02 and grown at 37°C to an OD_600_ value of 1.0. The cultures were pipetted into sterile 96-well flat-bottom tissue culture plates and incubated at 37°C for 1 h. The subsequent procedures were the same as those applied for the semi-quantitative biofilm assay.

### Immuno-dot blot analysis of PIA production

Immuno-dot blot assays were performed as described in our previous work [59]. PIA samples were isolated from the surface of cells in exponential growth phase (4 h) by boiling with 0.5 M EDTA. PIA production was determined by immuno-dot blot analysis using anti-PIA sera and quantified by photodigital analysis.

### Expression and purification of recombinant proteins

Different-length DNA fragments of the 2-kb coding region of *stk* were produced using genomic DNA of *S. epidermidis* as a template. The primers are listed in Table 2. The full-length PCR product of *stk* using primers 17F and 18R was digested with *Not*I and *Eco*RI and ligated into pET28a, yielding 6His-tagged protein (pET28a-Stk). As no tagged protein was expressed in pET28a, the *stk* fragment was acquired from pET28a-Stk plasmid digested with *Bam*HI and *Xho*I and subcloned into vector pGEX-KG, yielding pQG77 (WT Stk, Stk full length expression plasmid), allowing in-frame fusion with glutathione S-transferase (GST) [60]. Plasmids expressing GST fusions were constructed as follows: Stk codons corresponding to amino acid positions 1 to 263 (kinase domain, Stk^1–263^) were amplified using primers 59F and 60R introducing *Eco*RI and *Hind*III sites, and inserted into pGEX-KG (yielding pQG78). Stk codons corresponding to amino acid positions 40–263 (Stk kinase domain without ATP-binding site, Stk^40–263^) were amplified using primers 58F and 60R introducing *Eco*RI and *Hind*III and ligated into pGEX-KG (yielding pQG79). The resulting plasmids (Table 1) were used to chemically transform *E. coli* BL21 DE3 pLysS competent cells. The recombinant GST-tagged fusion proteins were expressed by the addition of 0.5 mM isopropyl-β-D-thiogalactopyranoside (IPTG) overnight at 16°C . The purification of the GST-tagged proteins were performed using GST-binding resin (Novagen) according to the manufacturer's instruction. Proteins were eluted with 20 mM glutathione prepared in 50 mM Tris-HCl (pH 8.0). Purified proteins were concentrated and dialyzed against PBS, using a 10 K and 30 K molecular weight dialysis cassette (Sangon Co.). Concentrations of proteins were determined using a Bradford Protein Assay kit (Sangon Co.) using bovine serum albumin as standard.

### *In vitro* kinase assay

For the *in vitro* kinase assay, 2 µg of WT Stk, Stk^1–263^ or Stk^40–263^ and 1 µg histone 1 (H1) were incubated separately in 50 µl kinase buffer (50 mM Tris-HCl [pH 7.5], 100 µM dithiothreitol [DTT], 1 mM MnCl_2_, 100 µM ATP) for 4 h at 32°C Kinase activity was assayed using a Kinase-Glo™ Luminescent Kinase Assay kit (Promega) and the luminescent signal reflecting the ATP level in the reaction system was measured in a Victor 3 plate reader. Staurosporine (Sangon Co.) was pre-incubated with WT Stk for 30 min at room temperature. The luminescent signal inversely correlates to the amount of kinase activity. All reactions were performed in triplicate.

### Quantitative real time polymerase chain reaction (RT-PCR)

*S. epidermidis* strains were grown in BM. Sample collection and RNA extraction were performed as described above. After treatment using a TURBO DNA-free™ kit (Ambion), approximately 2 µg of total RNA were reverse-transcribed with a PrimeScript RT reagent kit (TaKaRa). The cDNA was used as a template for real-time PCR using SYBR-green PCR reagents (TaKaRa). Reactions were performed in a MicroAmp Optical 96-well reaction plate using a 7500 Sequence Detector (Applied Biosystems). Primers used are listed in Table 2. All RT-PCR experiments were performed in triplicate, with gyrase B (*gyr*B) used as an internal control.

### Animal infection model and histopathological analysis

A murine subcutaneous foreign body infection model was performed as described by Kadurugamuwa et al [61]. Briefly, forty male BALB/c mice (Academia Sinica, weight ∼20 g) were randomly and evenly divided into two groups and infected with WT or *stk* mutant bacteria. One-centimeter long silicon catheters (14 gauge) were implanted subcutaneously on each side at the flank of each mouse before injection of 10^7^ CFU to the catheter bed. After ten days, the mice were sacrificed. Catheters were aseptically removed and manipulated with the method described previously except with a sonication time of 30 min. Then serial dilutions of the wash fluid were plated on TSB plates and the recovered *S. epidermidis* colonies were counted. For histological observations, the skin near the catheters were removed and immediately fixed in 2.5% (v/v) glutarildehyde-polyoxymethylene solution. These samples were embedded in paraffin and stained with hematoxylin and eosin (HE) and examined by light microscopy.

### Autolysis and bacteriolysis assays

Autolysis assays were performed using exponential-phase cells [13]. In brief, cells were cultured in TSB, harvested at an OD_600_ of 0.7, washed twice with ice-cold, sterile PBS, resuspended in PBS plus 0.1% Triton X-100, and incubated at 37°C with shaking at 250 rpm, and the optical densities were serially monitored. Bacteriolytic assays were performed as follows. Heat-killed cells were used as substrates and bacterial supernatants as the source of secreted autolysins [41]. Heat-killed cells were resuspended in PBS by heating the bacterial suspension at 65°C for 2 h and normalized to an OD_600_ value of 5.0. Secreted autolysins in the culture supernatants were normalized to cells at an OD_600_ value of 5, and sterilized by passage through a 0.22-µm filter. Bacteriolytic activity was assessed by mixing 1 ml of heat-killed cells with 5 ml of supernatant (or media as a control), incubation at 37°C with shaking at 250 rpm, and serial monitoring of the optical densities.
